# Acoustic recordings of underwater vocalizations of Indo-Pacific humpback dolphins in Xiamen Bay, China

**DOI:** 10.1038/s41597-025-06253-5

**Published:** 2025-12-18

**Authors:** Weijie Fu, Xuming Peng, Fuxing Wu, Fei Zhang, Chuang Zhang, Wenjie Xiang, Zhongchang Song, Yu Zhang

**Affiliations:** 1 Key Laboratory of Underwater Acoustic Communication and Marine Information Technology of the Ministry of Education, College of Ocean and Earth Sciences, Xiamen University, Xiamen, 361005 China; 2 Third Institute of Oceanography, Ministry of Natural Resources, Xiamen, 361005 China; 3 Observation and Research Station of Coastal Wetland Ecosystem in Beibu Gulf, Ministry of Natural Resources, Beihai, 536015 China

**Keywords:** Animal behaviour, Conservation biology

## Abstract

Vocalizations play crucial roles in dolphin biological activities. Analysis of dolphin vocalizations provides valuable insights into their behaviors and population status. In this data descriptor, we present a dataset of underwater vocalizations of Indo-Pacific humpback dolphins (*Sousa chinensis*) recorded in Xiamen Bay, China. The dataset comprises a diverse range of dolphin emissions, including 143 whistles (100 of which were classified as high-quality), and 897 pulse trains, categorized as echolocation clicks, burst pulses, and buzzes. A range of acoustic parameters was measured to characterize these acoustic signals. The presented dataset serves as an essential contribution to addressing existing data gaps regarding vocalizations of the Indo-Pacific humpback dolphin population in Xiamen Bay. It provides an important resource for studying vocalization patterns and temporal variability in the acoustic behaviors of the Indo-Pacific humpback dolphins, offering key insights to inform conservation strategies for this endangered population. Additionally, the dataset holds potential for population connectivity research, enabling acoustic comparisons between dolphin populations across different geographic regions to assess potential isolation or interaction.

## Background & Summary

To adapt to the complex and dark underwater environment, dolphins have evolved the ability to use sound for sensing their surroundings, navigation, foraging, and communication^1^. Dolphin vocalizations primarily consist of two main types: pulsed signals and frequency-modulated whistles. Pulsed signals, including echolocation clicks, burst pulses, and buzzes, are characterized by high-frequency, broadband properties and are typically produced in trains^2^. Among these, echolocation clicks are the most commonly observed acoustic signals. Dolphins emit highly directional echolocation clicks while navigating and detecting prey or other targets of interest^2^. Burst pulses share similar characteristics with echolocation clicks but generally have lower frequencies and higher repetition rates. These signals are primarily used for intraspecific communication^3^. Buzzes are associated with close-range echolocation, particularly during the capture phase of hunting, as indicated by a rapid increase in pulse repetition rate. In addition to their role in predation, buzzes also serve as functional signals in social interactions, such as mating behaviors and mother-calf communication^4^. Whistles, which are narrow-band signals with modulated frequencies, are predominantly used for communication^1^. This type of emission plays crucial roles in social contexts, including reproductive gathering^5^, group cohesion^6^, individual identification^7^, coordinating group activities^8^.

Dolphin vocalizations, both whistles and pulsed signals, are highly dynamic and flexible. The characteristics of dolphin sound production are known to vary depending on the environmental conditions^9–15^. For instance, Jensen *et al*.^9^ demonstrated that Irrawaddy dolphins (*Orcaella brevirostris*) and Ganges river dolphins (*Platanista gangetica gangetica*) produce echolocation clicks with significantly lower peak frequencies and sound amplitudes in shallow waters compared to deep waters, likely to mitigate reverberation. The minimum, maximum, and peak frequencies of whistles produced by common bottlenose dolphins (*Tursiops truncatus*) were found to increase with the ambient noise^10^. Changes in both click and whistle parameters in response to vessel presence have also been reported^11–14^. Additionally, alterations in dolphin sound production patterns have been linked to factors such as group size, group composition, and behavioral contexts^12,15–17^. For example, Akiyama and Ohta^18^ observed that common bottlenose dolphins increase whistle production during feeding, with a preference for upsweep-contour whistles. In dolphin groups containing calves, whistles tend to have lower end and minimum frequencies and longer durations^12^. Dolphins also exhibit adaptive modifications in their click characteristics during prey perception and target detection. They dynamically adjust their click rate, output amplitude, and acoustic directivity across different phases of target approach to achieve precise detection and recognition^19–22^. Recently, research on the adaptive acoustic control of dolphin biosonar during target detection has intensified, driven by the dual objectives of understanding biological systems and inspiring biomimetic applications.

In summary, dolphin vocalizations are complex and dynamic yet critical for their survival. The collection and analysis of these acoustic signals can help to better understand their behaviors and population status. As one of the vulnerable dolphin species assessed by the IUCN^23^, the Indo-Pacific humpback dolphin (*Sousa chinensis*) in Chinese waters is sporadically distributed along the southeastern coast of China, including Xiamen Bay. The population inhabiting Xiamen Bay is under significant survival pressure due to increasing anthropogenic disturbances, with a notable reduction in population size over the past two decades^24–30^. Enhanced conservation efforts are urgently needed to mitigate ongoing threats to this endangered population. A comprehensive assessment of population status, including population size and structure, distribution patterns, surface behaviors, and vocalizations, is essential for developing effective conservation strategies and management initiatives. While studies have reported on vocalizations of Indo-Pacific humpback dolphins in Chinese waters^31–39^, limited research focused on the Xiamen Bay population, necessitating more targeted bioacoustic investigations.

In this paper, we present a dataset of acoustic recordings of Indo-Pacific humpback dolphin vocalizations, collected during our regular surveys of the species’ population status. These data represent an important supplement to the Indo-Pacific humpback dolphin sound library in Chinese waters and, to a large extent, fill the data gaps regarding the dolphin population in Xiamen Bay. In addition to sharing the complete acoustic data, a quantitative analysis was conducted on dolphin vocalizations, both whistles and pulsed signals (classified as echolocation clicks, burst pulses, and buzzes), to determine characteristic parameters of each signal, and the results are incorporated into the dataset. It offers a valuable resource to study the vocalization patterns of the Indo-Pacific humpback dolphin population in Xiamen Bay, as well as to investigate population connectivity through acoustic comparisons across different geographic regions, helping to assess potential population isolation or interaction. Notably, this dataset spans a period of up to three years, allowing for investigations into potential temporal variability in dolphin acoustic behaviors over a long timeline. The Indo-Pacific humpback dolphins inhabit shallow coastal waters where they face significant echolocation challenges due to high reverberation. Dolphins present remarkable biosonar plasticity, adjusting their acoustic signals in response to varying environmental conditions^9–15^. This dataset contains echolocation click trains recorded across a wide range of water depths from 2.1 m to 24.3 m, providing valuable acoustic materials for exploring biosonar operational mechanisms— particularly how dolphins adapt their signals in shallow-water environments with strong reverberation. The comprehensive collection of dolphin vocalization signals also serves as an important resource for bionic applications^40–43^.

## Methods

### Data collection

**Fig. 1:**
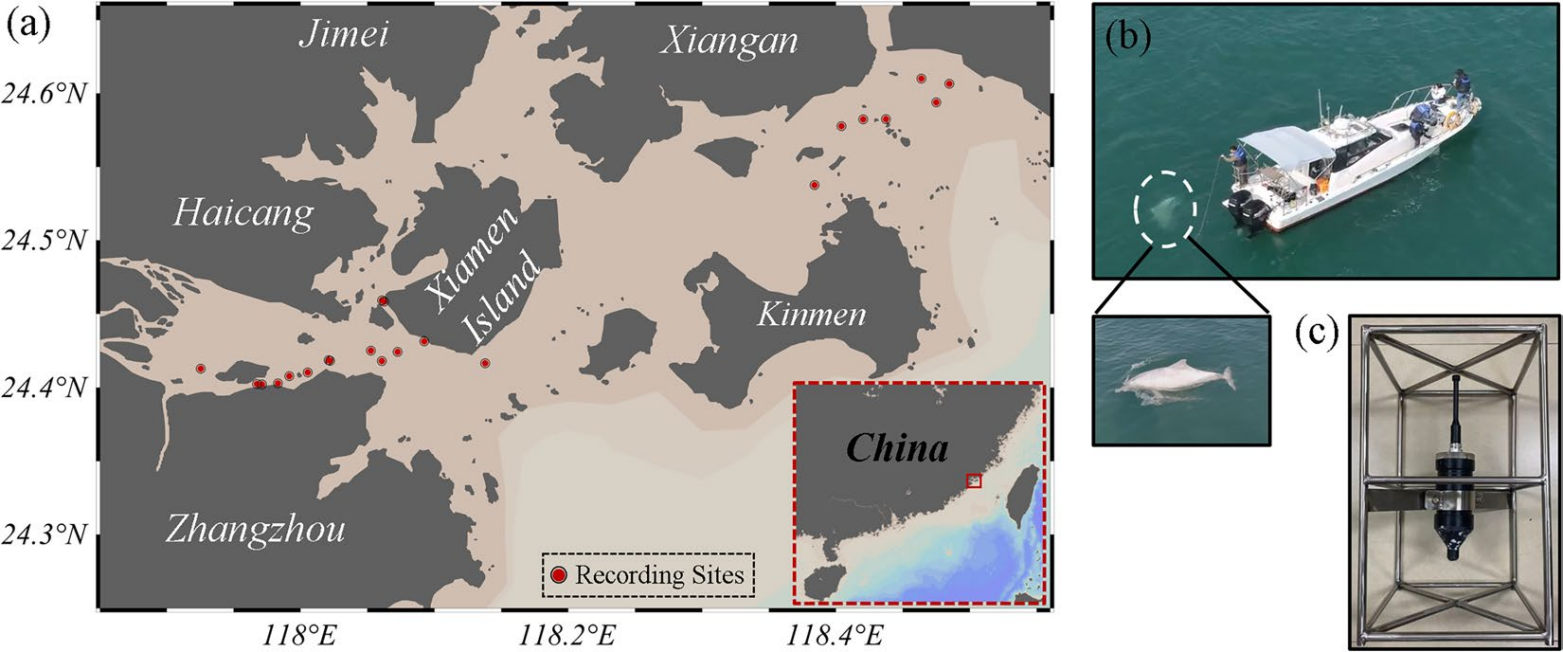
(**a**) Map of the survey area, with red dots indicating locations where acoustic recordings of dolphin vocalizations were conducted. (**b**) Aerial photographs of the survey vessel and an Indo-Pacific humpback dolphin captured by an unmanned aerial vehicle. (**c**) The SoundTrap 300HF underwater acoustic logger used in the survey.

We conducted a total of 22 regular vessel-based surveys in Xiamen Bay, China, from June 2022 to September 2024, to investigate the abundance and distribution patterns of the Indo-Pacific humpback dolphin population, as well as their underwater vocalizations (Fig. 1). During the surveys, the vessel navigated at a speed of 5–7 knots, with three observers equipped with Navigator 7 × 50 binoculars (magnification: 7×, objective lens diameter: 50 mm, field of view: 419 ft at 1000 meters, Steiner company, Germany) positioned on the forward deck to search for dolphin presence. Upon sighting dolphins, the vessel approached at a reduced speed and stopped its engine once a distance was reached less than 50 m. During this period, a calibrated underwater acoustic recorder, SoundTrap 300HF (Ocean Instruments, Auckland, New Zealand), was deployed to record the underwater sounds produced by the dolphins. The recorder was equipped with a 16-bit analog-to-digital converter (ADC) and a pre-amplified hydrophone exhibiting a linear frequency response across the frequency range from 20 Hz to 150 kHz, with a sensitivity of −189 ± 3 dB (re 1 V/μPa) in low-gain mode. The recorder was housed in a steel holder and positioned 1.5–2 m underwater using a steel pipe. A sampling rate of 576 kS/s was used, and the recorded sound data were stored as WAV audio files in real time. During acoustic recording, the vessel engine was turned off to minimize low-frequency sound interference. Dolphins were photographed for individual identification, and detailed information about each acoustic recording session was documented, including geographic location, water depth, dolphin group size, and dolphin behavioral state. From our field surveys, a total of 1019 minutes of original sound data were collected. These recordings were subsequently processed to extract communication whistles and high-frequency pulsed signals produced by the Indo-Pacific humpback dolphin.

### Detection of whistles

**Fig. 2:**
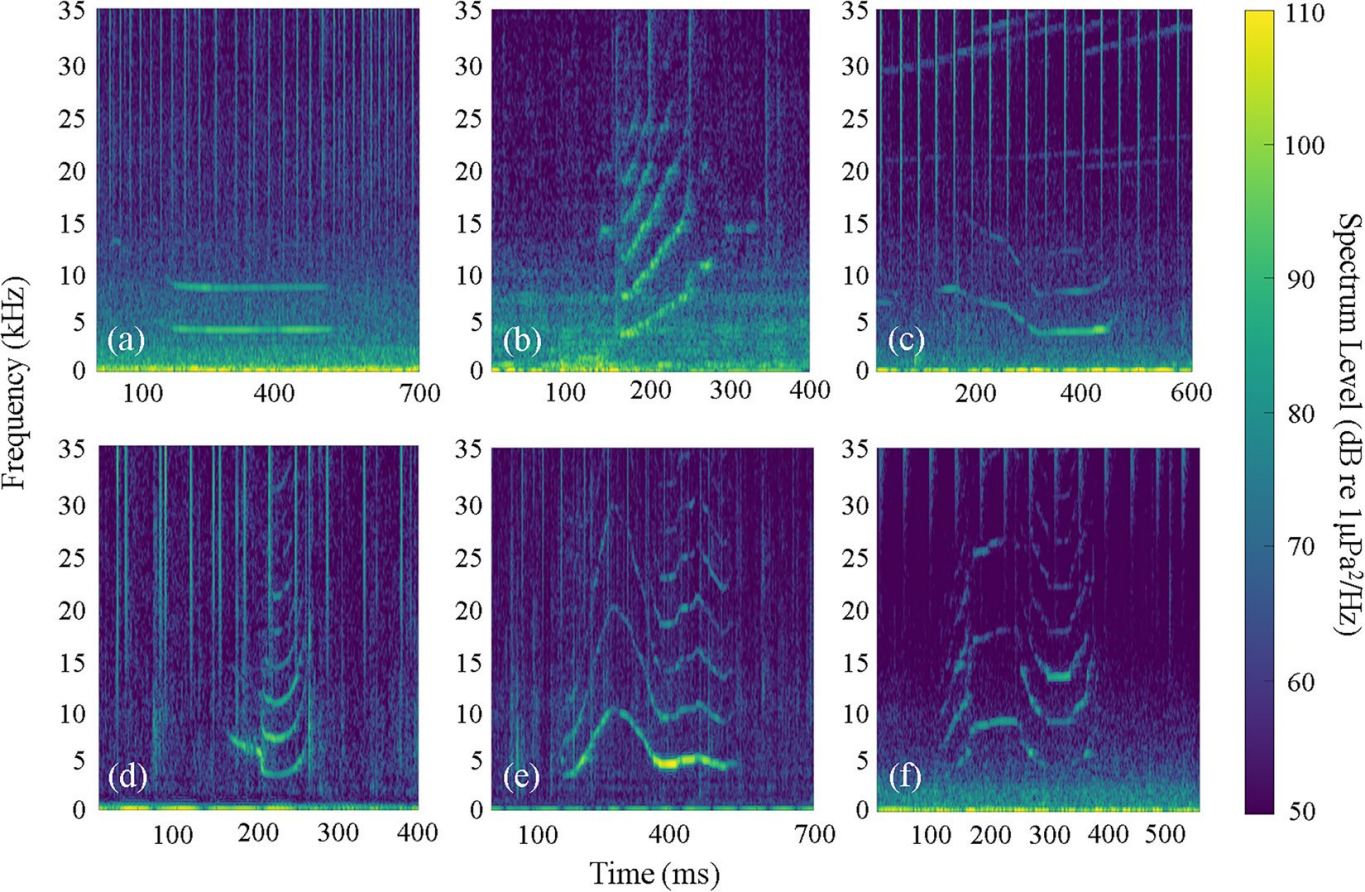
Example frequency contours illustrating the six whistle types: (**a**) constant, (**b**) upsweep, (**c**) downsweep, (**d**) concave, (**e**) convex, (**f**) sinusoidal.

The original WAV files were displayed in the time-frequency domain using Adobe Audition (Version 2021) and manually reviewed by an experienced observer to identify whistles within consecutive 3-second time windows. A continuous tonal contour without temporal breakpoints on the spectrogram was identified as a single whistle. Additionally, consecutive contours were also considered a single whistle if the gap between them was shorter than 200 ms and less than the duration of the contours, following established methodologies^35,44^. Each identified whistle signal was subsequently extracted and saved as an individual WAV file, and the number and position of whistles within the original acoustic file were documented. Whistles were visually divided into three grades based on signal-to-noise ratio (SNR), referencing previous studies^12,45^. Grade 1 includes whistles with weak contour, but is visible in the spectrogram; Grade 2 includes whistles with clear and unambiguous contours; and Grade 3 includes whistles with contour prominent in the spectrogram. Whistles of Grade 2 and 3 were considered to be of high quality and selected for detailed analysis. All whistle contours were visually categorized into six tonal types^32,35,36,44^ (Fig. 2): (a) constant, (b) upsweep, (c) downsweep, (d) concave, (e) convex, and (f) sinusoidal. For each high-quality whistle, 13 acoustic characteristics were manually measured from the spectrograms: (a) duration (ms), (b) start frequency (kHz), (c) end frequency (kHz), (d) frequency change (kHz), (e) absolute frequency gradient (kHz/s), (f) minimum frequency (kHz), (g) maximum frequency (kHz), (h) delta frequency (kHz), (i) number of extrema, (j) number of inflection points, (k) number of saddle points, (l) number of breaks, and (m) presence/absence of harmonics. Detailed definitions of these acoustic parameters were consistent with those described in Marley *et al*.^45^.

### Detection of pulsed signals

The raw acoustic data contained various pulsed sounds produced by the Indo-Pacific humpback dolphins, including echolocation clicks, burst pulses, and buzzes. These pulsed signals have been shown to exhibit a Gabor-type waveform structure^46,47^, characterized by a distinct Gaussian envelope in the output of the Teager–Kaiser energy operator (TKEO). Based on this feature, a mature processing method proposed by Madhusudhana *et al*.^48^ was applied to extract pulsed signals from the recordings. Initially, raw WAV files were split into 30-second segments to reduce the computational load. A Butterworth high-pass filter with a cut-off frequency of 5 kHz was used to remove most low-frequency noise from the acoustic data.

Subsequently, the TKEO output was calculated for each filter data segment as follows^49^:

1 $${\Psi }_{d}[{x}_{n}]={x}_{n}^{2}-{x}_{n-1}{x}_{n+1}$$

where x_n_ represents the sampled points of each 30-second signal segment. A Gaussian-weighted averaging filter (MAF1) was then applied to highlight short-duration energy surges in TKEO outputs. The Gaussian filter was defined as:

2 $$MAF1(n)=\frac{{T}_{s}}{{\sigma }_{G}\sqrt{2\pi }}{e}^{-{(n{T}_{s})}^{2}/2{{\sigma }_{G}}^{2}}$$

where *n* = *−N, …, 0, …, N*, is the index of the sampled point in the filter, *T*_*s*_ is the sampling interval, and ${\sigma }_{G}$ is the standard deviation of the Gaussian, given by:

3 $${\sigma }_{G}=\frac{FWHM}{2\sqrt{2\,\mathrm{ln}(2)}}$$

where FWHM is the width of the Gaussian at half its peak value, which is taken as 1 × 10^−4^ s, based on the length of a representative pulsed signal produced by the dolphin.

The value of N is determined based on ${\sigma }_{G}$, given as the following formula:

4 $$N=\frac{5{\sigma }_{G}}{{T}_{s}}$$

where *T*_*s*_ is the sampling interval.

Convolution of TKEO output with MAF1 can be expressed as:

5 $${h}_{MAF1}(n)=\frac{{T}_{s}}{{\sigma }_{G}\sqrt{2\pi }}\mathop{\sum }\limits_{i=-N}^{N}{e}^{-{(i{T}_{s})}^{2}/2{{\sigma }_{G}}^{2}}{x}_{n+i}$$

TKEO outputs were also filtered by a rectangular averaging filter (MAF2) with identical length and filter gain to MAF1:

6 $$MAF2(n)=\frac{\mathop{\sum }\limits_{m=-N}^{N}MAF1(m)}{2N+1}$$

The filter difference ratio (FDR) was then computed to measure the difference in the responses of the two filters:

7 $$FDR(n)=\frac{{h}_{MAF1}(n)-{h}_{MAF2}(n)}{{h}_{MAF1}(n)}$$

**Fig. 3:**
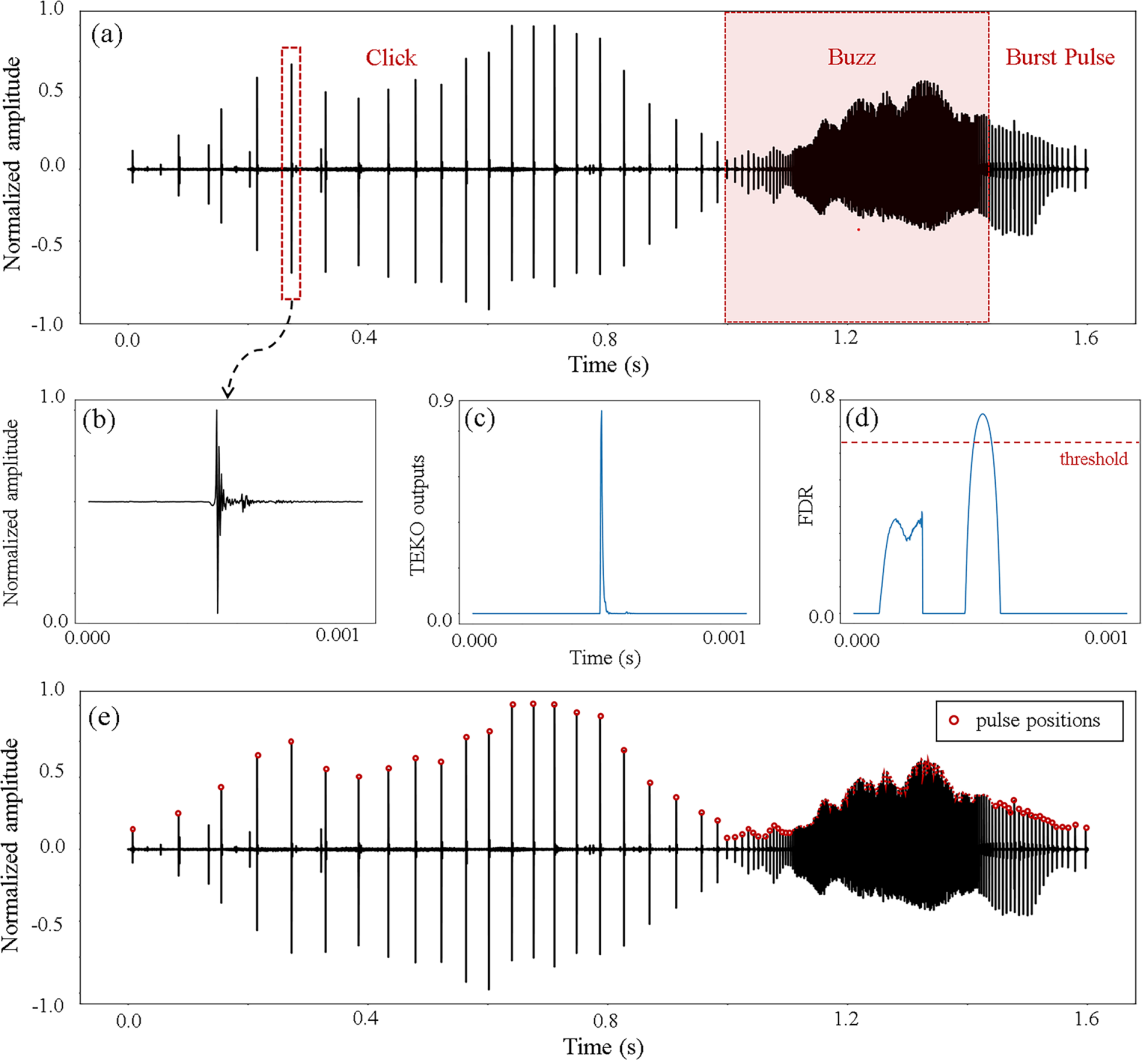
(**a**) Example data segment of dolphin vocalizations containing clicks, buzzes, and burst pulses. (**b**) A representative single pulsed signal, along with its outputs from (**c**) the Teager–Kaiser energy operator (TEKO) and (**d**) the filter difference ratio (FDR). (**e**) Positions of dolphin pulsed signals within the data segment as identified by the automated processing procedure.

The obtained FDR is expected to yield local maximums at locations of Gaussian-like spikes in the TKEO outputs, indicating the presence of pulsed signals in the original recordings (Fig. 3). Since the FDR values remains relatively constant irrespective of signal amplitude, a detection threshold was set at 85% of the peak FDR value (FDR_peak_) following Madhusudhana *et al*.^48^ Pulsed signals were confirmed only when the FDR value exceeded this threshold and the signal-to-noise ratio (SNR) of the signal was above 10 dB. For all identified pulses, the inter-pulse intervals (IPIs) were measured, which is defined as the time interval between two consecutive pulses.

Pulse trains were identified by using an adaptive IPI threshold^50^. Consecutive pulses with a gradual change in the IPI were recognized to belong to the same pulse train, and a pulse train was considered terminated when an abrupt IPI increase occurred. Each pulse train detected was visually confirmed, and those containing signals with high reverberation and from more than one animal were removed. Based on IPI thresholds established by Wang *et al*.^37^, the identified pulse trains were subsequently classified into echolocation clicks, burst pulses, and buzzes, according to their mean IPI values. Pulse trains with a mean IPI value less than 4.9 ms were recognized as buzzes, those with mean IPIs greater than 15.5 ms were considered echolocation clicks, and pulse trains with intermediate mean IPIs (4.9–15.5 ms) were categorized as burst pulses. Each identified pulse train, along with its constituent individual pulses, were saved as separate WAV files within the dataset. Pulse trains were sequentially numbered based on their chronological order of appearance in the original recordings, and the position and total number of pulse trains in each original recording were documented. For each identified pulse train, six acoustic parameters were measured for the pulsed signals according to previous studies^2,51,52^: (a) inter-pulse-interval (IPI), (b) sound pressure level (SPLpp), (c) duration, (d) peak frequency (Fpeak), (e) -3dB bandwidth (-3dBBW), and (f) -10dB bandwidth (-10dBBW). Additionally, the mean values of these acoustic parameters were calculated and recorded for each pulse train.

## Data Records

The acoustic dataset contained a total of 143 whistle signals, of which 100 were identified as high-quality based on visual inspection. Additionally, 897 pulse trains were included, comprising 832 echolocation click trains, 15 burst pulse trains, and 50 buzz trains. The complete dataset is publicly accessible via an unrestricted repository at figshare^53^, consisting of WAV audio files, TXT text document files, Excel data sheets, and Portable Network Graphic (PNG) image files.

The acoustic recordings are provided in different folders based on the signal type:

1. **Original Audio File** contains the complete original recordings collected from field surveys, including 35 WAV audio files sequentially named according to the recording time (e.g., Ori_Recording_01.wav, Ori_Recording_02.wav, Ori_Recording_03.wav).
2. **Whistles** comprises 143 extracted whistle signals saved as separate WAV files (e.g., Whistle_001.wav, Whistle_002.wav, Whistle_003.wav), along with PNG image files illustrating spectrograms of each whistle (e.g., Whistle_001.png, Whistle_002. png, Whistle_003.png).
3. **Click Trains, Burst Pulse Trains, and Buzz Trains** contain WAV audio files corresponding to the detected click trains, burst pulse trains, and buzz trains, respectively. Each pulse train, along with its individual pulsed signals, is saved in a separate subfolder. These subfolders are sequentially named according to the serial number of the pulse train (e.g., PulseTrain_001, PulseTrain_002, PulseTrain_003). Each subfolder additionally contains a tab-separated TXT file named “**PulseParameters.txt**”, documenting acoustic parameters for each pulsed signal.

**Results.xlsx** is an Excel file containing detailed information on the original acoustic recordings and quantitative data on whistles and pulsed signals detected within each original acoustic file:

1. recording dates when the original acoustic data were collected;
2. geographic coordinates (latitude and longitude) of the recording locations;
3. start and end times of each original acoustic file;
4. number of pulse trains detected within each original acoustic file;
5. number of identified whistles, including counts of high-quality whistles (Grade 2 and 3) within each original acoustic file;
6. additional information about the recordings, including season, tide, water depth, dolphin group size, and behavioral state of dolphins.

**Whistles.xlsx** is an Excel file that provides detailed descriptions of each whistle signal:

1. the original acoustic file from which the whistle signal was extracted;
2. start and end times of each whistle signal within the original acoustic file, relative to the file’s start, expressed in seconds;
3. contour type classification for each whistle signal (constant, upsweep, downsweep, concave, convex, and sinusoidal);
4. quality grading for each whistle (Grade 1, Grade 2, and Grade 3);
5. acoustic parameters of each whistle signal: Duration, Start Frequency, End Frequency, Frequency Change, Absolute Frequency Gradient, Minimum Frequency, Maximum Frequency, Delta Frequency, Number of Extrema, Number of Inflection Points, Number of Saddle Points, Number of Breaks, and Presence/Absence of Harmonics.

**ClickTrains.xlsx, BurstPulseTrains.xlsx, and BuzzTrains.xlsx** are Excel files containing detailed information on each identified pulse train type:

1. serial number of pulse trains denoting their order of appearance within original recordings;
2. original acoustic file from which each pulse train was extracted;
3. start and end times of each pulse train within the original acoustic file, relative to the file’s start, expressed in seconds;
4. lengths of each pulse train (Length);
5. number of pulsed signals within each pulse train (NumP);
6. mean values of acoustic parameters of the pulsed signals within each pulse train: mean SNR (Mean_SNR), mean IPI (Mean_IPI), mean sound pressure level (Mean_SPLpp), mean duration (Mean_Duration), mean peak frequency (Mean_Fpeak), mean -3dB bandwidth (Mean_-3dBBW), and mean -10dB bandwidth (Mean_-10dB BW).

## Technical Validation

To ensure the reliability of the presented dataset, the signal processing procedures in this study strictly adhered to the methodologies reported in published studies^32,35,36,44,45,48,54,55^. The identification, quality grading, classification, and subsequent characteristic measurements of the whistles were manually completed and reviewed by trained and experienced operators using the specialized acoustic signal processing software (Adobe Audition, Version 2021). Statistical results for the whistle parameters are summarized in Table 1, including the range (minimum to maximum), mean, and standard deviation for the 12 measured characteristic parameters of the 100 high-quality whistles.

**Table 1:** Summary statistics of the characteristic parameters for the 100 high-quality whistles.

Characteristic Parameter	Range (Min - Max)	Mean ± Sd
Duration (ms)	21.4–891.6	315.2 ± 203.5
Start Frequency (kHz)	2.49–12.71	5.37 ± 2.08
End Frequency (kHz)	0.88–13.80	5.49 ± 2.08
Frequency Change (kHz)	0–7.33	1.18 ± 1.53
Absolute Frequency Gradient (kHz/s)	0–84.11	6.88 ± 14.44
Minimum Frequency (kHz)	0.88–12.39	4.73 ± 1.87
Maximum Frequency (kHz)	3.32–14.32	6.70 ± 2.50
Delta Frequency (kHz)	0–10.44	2.14 ± 2.06
No. Local Extrema	0–3	0.5 ± 0.7
No. Inflection Point	0–3	0.5 ± 0.8
No. Saddle Point	0–5	0.3 ± 0.6
No. Break	0–3	0.4 ± 0.7

**Fig. 4:**
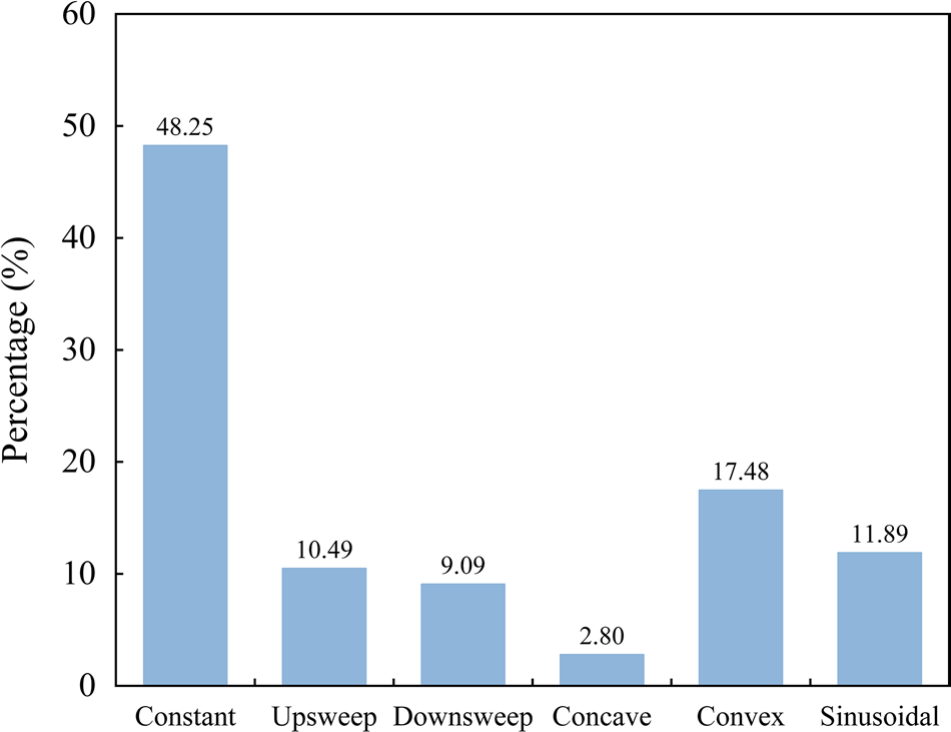
Proportions of the six contour types in all whistles in the dataset.

Local extrema, inflections, saddles, breaks, and harmonics were observed in 56%, 36%, 30%, 42%, and 65% of all high-quality whistles, respectively. The constant-shaped contour was the most dominant tonal type, accounting for 48.25% of all whistles identified in this dataset (Fig. 4), consistent with previous reported data for Indo-Pacific humpback dolphin populations in Zhanjiang, Sanniang Bay, and Hainan waters^32,35,36^. This consistency provides support for the reliability of the presented dataset.

Detection and classification of pulsed signals were conducted following standard procedures and methodologies widely adopted in published research^54–60^. Clicks were the most frequent pulse type, comprising approximately 93% of the identified 897 pulse trains, with a total of 832 occurrences. The acoustic characteristics of the three pulse train types are statistically summarized in Table 2, and each parameter was averaged per train. Consistent with previous findings^37^, the pulsed signals in this dataset were characterized by high-frequency, broadband features, with click trains exhibiting longer train lengths and higher peak frequencies compared to burst pulses and buzzes. Among the three sound types, burst pulses had the lowest peak frequency and bandwidth. These results further support the reliability of the presented dataset.

**Table 2:** Descriptive statistics for acoustic characteristics of pulse trains produced by Indo-Pacific humpback dolphins in Xiamen Bay, China.

Train Type	Statistics	Length (ms)	NumP	IPI (ms)	Duration (μs)	Fpeak (kHz)	-3dBBW (kHz)	-10dBBW (kHz)
Click (n = 832)	Mean ± Sd	1239.68 ± 1218.60	22.1 ± 24.4	80.84 ± 60.38	71.83 ± 22.68	87.48 ± 18.78	19.62 ± 9.25	72.55 ± 23.78
	Min-Max	33.07–11260.44	3–229	15.69–368.77	21.73–144.10	25.12–147.00	6.58–72.14	11.81–142.93
Burst pulse (n = 15)	Mean ± Sd	516.36 ± 388.49	48.6 ± 32.1	10.61 ± 3.24	90.77 ± 28.33	51.65 ± 14.06	20.16 ± 8.99	57.88 ± 19.28
	Min-Max	11.27–1054.68	3–93	5.45–15.41	54.61–160.02	29.51–74.78	7.31–38.68	27.99–97.94
Buzz (n = 50)	Mean ± Sd	117.44 ± 165.62	48.5 ± 45.4	2.13 ± 0.73	88.01 ± 19.89	72.82 ± 15.35	15.68 ± 5.80	76.25 ± 23.28
	Min-Max	9.44–924.97	6–203	1.42–4.47	39.16–136.66	36.77–99.25	7.5–31.47	16.53–128.87

Note that each parameter was averaged per train.

## Data Availability

The acoustic dataset described in the current paper is publicly accessible via an unrestricted repository at figshare (10.6084/m9.figshare.29143727).

## Code availability

Data processing followed established published methodologies, and no custom code was used in this paper. For detailed procedures on pulsed signal detections, refer to Madhusudhana *et al*.^44^.
